# Power and Radio Resource Management in Femtocell Networks for Interference Mitigation

**DOI:** 10.3390/s21144843

**Published:** 2021-07-15

**Authors:** Sultan Alotaibi, Hassan Sinky

**Affiliations:** 1 Department of Information Systems, Umm Al-Qura University, Makkah City 24381, Saudi Arabia; 2 Department of Computer Science, Umm Al-Qura University, Makkah City 24381, Saudi Arabia; hhsinky@uqu.edu.sa

**Keywords:** femtocells, interference mitigation, resource allocation, transmission power control

## Abstract

The growth of mobile traffic volume has been exploded because of the rapid improvement of mobile devices and their applications. Heterogeneous networks (HetNets) can be an attractive solution in order to adopt the exponential growth of wireless data. Femtocell networks are accommodated within the concept of HetNets. The implementation of femtocell networks has been considered as an innovative approach that can improve the network’s capacity. However, dense implementation and installation of femtocells would introduce interference, which reduces the network’s performance. Interference occurs when two adjacent femtocells are operated with the same radio resources. In this work, a scheme, which comprises two stages, is proposed. The first step is to distribute radio resources among femtocells, where each femtocell can identify the source of the interference. A constructed table is generated in order to measure the level of interference for each femtocell. Accordingly, the level of interference for each sub-channel can be recognized by all femtocells. The second stage includes a mechanism that helps femtocell base stations adjust their transmission power autonomously to alleviate the interference. It enforces a cost function, which should be realized by each femtocell. The cost function is calculated based on the production of undesirable interference impact, which is introduced by each femtocell. Hence, the transmission power is adjusted autonomously, where undesirable interference can be monitored and alleviated. The proposed scheme is evaluated through a MATLAB simulation and compared with other approaches. The simulation results show an improvement in the network’s capacity. Furthermore, the unfavorable impact of the interference can be managed and alleviated.

## 1. Introduction

The exponential growth of mobile traffic volume has become one of the critical issues in wireless communication networks. As a result, the overall network capacity degradation has been considered one of the significant challenges of future wireless communication networks. Therefore, ultra-dense heterogeneous networks (HetNets) have been proposed as a promising approach to support the dramatic increase of wireless data and improve user experience [1,2,3]. The ultra-dense HetNets can significantly improve capacity and spectral efficiency with low transmission power resulting from enhanced frequency reuse [4,5,6]. In HetNets, small cells with short distance transmission capability are installed and superimposed on the macrocells’ coverage area. There are three different types of small cells: microcell, picocell, and femtocell. They are categorized based on their coverage and radius. The femtocell networks are considered in this work.

HetNets based on ultra-dense deployment of small cells in a multi-tier architecture are the solution to meet future cellular networks’ capacity and data rate requirements. The deployment of small cells in HetNets provides efficient traffic offloading between network tiers to efficiently promote the growing mobile traffic with improved QoS and increased data rates. Small cells such as a femtocells, which have low power transmission with a short coverage area, can operate in both a licensed and unlicensed spectrum. In femtocells, the transmitter and receiver come closer, resulting in a decreased cell radius and increase in coverage and connectivity compared to the traditional macrocell base station. However, there is a trade-off between femtocell capacity and coverage area with energy consumption and construction cost.

One of the preferred ideas that revolutionized the cellular network is known as the femtocell base station (FBS). The concept of FBS networks is considered as an enabler of the future generation mobile network, which requires extremely high data rate links. Moreover, FBS will be used to offload traffic from the current mobile generation network. Therefore, it seems to be a promising solution regarding the issue of increasing generated traffic in terms of indoor environments. Additionally, it is expected that the indoor traffic will exponentially increase in the near future. Therefore, the macrocell base station may experience challenges such as congestion because of the increased traffic.

**Figure 1:**
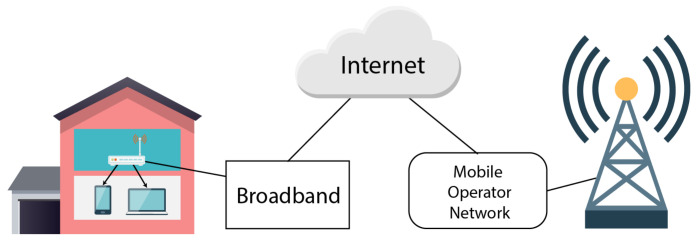
FBS installation within MBS coverage area.

The FBS is a low power transmission base station. In addition, in terms of deployment, it is relatively inexpensive. Moreover, the end user deploys it, and it is connected to the customer’s backhaul connection [7]. The installation of the FBS is overlayed on macrocell MBS dominant coverage, as depicted in Figure 1. Via the Internet connection, the femtocell is connected with the macrocell, so the data are transmitted securely. This link is needed in order to establish a secure communication channel between a macrocell and its femtocells. Performing functions such as handover between macrocells and femtocells requires coordination between femtocells and macrocells.

Indeed, introducing femtocells strengthens the network’s coverage. The radius of the femtocell should not exceed 50 m. Accordingly, receiving signals in short distance has less impact on the battery of the UEs. Additionally, the throughput can be increased. A macrocell is also able to offload more UEs to femtocells. Such a procedure improves the capacity of all attached UEs. The femtocell network architecture consists of three main entities: femtocells, femtocell user equipment (FUE), and the femtocell management system (FMS). Femtocells are the deployed base stations, and they have most of the macrocell functionalities. FUEs are the end users who are connected to the femtocell base stations. The FMS entity is used to link femtocells to the Internet with the backhaul connection and controls the group of femtocells, which is deployed in a certain geographical area [8].

Furthermore, the femtocell can operate in one of the following three access modes. In the closed access mode, only a specific group of UEs can access the femtocell. The open access mode is considered when there are no restrictions for accessing the femtocell. In the hybrid access mode, accessing the network can be available for any UEs, but in some cases, preferences would be given to those who subscribe to a specific femtocell [9,10,11]. Although femtocells are deployed in the range of a particular macrocell, novel mechanisms need to be designed to ensure that this joint deployment exists simultaneously. For example, it would be preferable to access the available spectrum for the UEs of a macrocell. Femtocells are, therefore, necessary not to breach the consistency of this coexistence. The femtocell network architecture subsequently follows two modes of service to access the shared spectrum: underlay and overlay. In the underlay scenario, the macrocell becomes a primary system where femtocells are recognized as a secondary system. In this scenario, the priority goes to the primary system over the secondary system, and the secondary system should ensure that the predefined restrictions are not violated. In the overlay scenario, macrocells and femtocells should share the spectrum based on a cooperative manner. Moreover, femtocells can be used for private purposes or enterprise purposes [12,13,14,15].

Femtocells are deployed as an extension of traditional cellular networks, especially for dead zones and indoor environments. As a result, several technical challenges will occur when femtocells are deployed and overlaid in the dominant macrocell region. Therefore, these issues need to be identified and tackled in order to strengthen the functioning of femtocell networks. Challenges evolve pertaining to, but not limited to, guidelines on access modes, managing handover activities, resource allocation, spectrum sharing systems, interference management, and control of resource policy [16].

Although the massive deployment of femtocells enhances the network’s capacity, some challenges arise. Indeed, the deployment of femtocells is controlled inefficiently because the end user is in charge of installing femtocells; therefore, dense and unplanned femtocell deployment limits the planned advantages of using femtocells. Hence, the key challenge of deploying femtocell networks is interference. Interference occurs when the same frequency of radio resources is allocated to different UEs, which are associated with different femtocells, and they are found in overlapping areas. The interference causes degradation in the overall network performance and decreases the network efficiency. Interference is one of the issues that needs to be tackled effectively. Moreover, interference management is considered to be a crucial obstacle for the efficient implementation of two-tier networks, such as the deployment of femtocell networks across a particular conventional macrocell area [17]. There are usually two forms of interference: cross-tier interference and co-tier interference. Those forms of interference fundamentally exist in two-tier networks. When both nodes, the source and the victim, are classified in the same network tier, co-tier interference occurs. Consequently, a serious co-tier interference occurs when a large number of femtocells are densely deployed, and the problem becomes difficult to deal with. Cross-tier interference, on the other hand, happens when the source and victim nodes belong to different networks [18,19,20]. Table 1 summarizes all possible types of interference.

Despite the different benefits of the deployment of femtocells, deployment cost, network reliability, and interference are real concerns. A significant challenge for improving femtocell networks is interferences between and within tiers, known as cross-tier and co-tier interferences.

The major contributions of this paper are summarized as follows:

- We proposed a scheme to address the problem of interference and to improve the capacity of the femtocells’ network. The proposed scheme has two major stages.
- The first stage is to control the distribution of the radio resources among femtocells. In this stage, we adopted the concept of fractional frequency reuse (FFR). The FFR concept is used to divide the coverage of a certain macrocell into four major sub-areas. Then, the spectrum will also be divided into four sub-bands according to the division of the macrocell’s coverage. Then, the proposed scheme can construct an interference-based table to manage the radio resource distribution among femtocells according to Section 4 in this work.
- The second stage of the proposed scheme is to control the transmission power for each resource block (RB) to address the interference in terms of power control. This stage starts with calculating the level of the interference, which is introduced by each femtocell. Accordingly, the transmission power can be autonomously adjusted to alleviate the impact of undesirable interference.
- We conduct a MATLAB simulation to evaluate the proposed scheme. In addition, the proposed scheme was compared to existing approaches.

## 2. Related Work

Rapid urbanization and network infrastructure densification coupled with recent mobile data usage trends have led to an unprecedented increase in wireless devices, services, and applications, with varying quality-of-service (QoS) needs and requirements in terms of latency, data rates, and connectivity. This proliferation of wireless/Internet devices and our insatiable demand for wireless connectivity have led to an exponential growth in network capacity demand. As a result, ultra-dense heterogeneous networks (HetNets) have adopted a number of newly emerging wireless technologies, such as dynamic bandwidth sharing and mmWave spectrum access, to help cope with these rising demands. In turn, the introduction of HetNets has allowed for expanded data rates, increased network capacity and spectrum reuse, and improved QoS provisioning.

However, these integrating technologies in HetNets often involve many challenges and constraints. That is, traditional wireless issues, such as network capacity utilization, energy efficiency, interference mitigation, and dynamic spectrum access, are further exacerbated with ultra-dense HetNets. For instance, the ultra-dense characteristics of HetNets induce high computational complexity considerably due to the involvement of large numbers of users and network access points, where multidimensional resource allocation occurs in the frequency, time, power, and space domains. In addition, the difficulty in obtaining global channel, queue, and cache state information incurs significant signaling overhead. Furthermore, new applications and services require new content- and computation-intensive and delay-sensitive QoS demands. Finally, fast access and seamless handoffs are essential in densely deployed environments where mobile users find themselves frequently seeking the optimal QoS, which further complicates the task of resource allocation.

Thus, designing a robust resource allocation technique that accounts for these issues is a considerable challenge. Many resource allocation schemes employ techniques to maximize network capacity or minimize power consumption yet overlook the undesirable impact that interference has on the aforementioned advantages of HetNets. In ultra-dense deployments, the interference problem becomes rather serious and difficult to alleviate as many heterogeneous access points are naturally in close proximity. Hence, efficient power and radio resource management algorithms are essential for interference mitigation and QoS provisioning.

The impact of the interference on the overall networks’ capacity is unfavorable. Therefore, a mechanism is needed to address the unfavorable impact of the interference on the networks’ capacity. Abundant proposed solutions and schemes have previously been introduced to address the interference challenge. Suggested mechanisms handle the interference based on various aspects. Some mechanisms are proposed based on controlling the power transmission of the femtocells and their SINR. In [21], the authors proposed an algorithm to mitigate the undesirable impact of the femtocell’s co-channel interference. They suggested an SINR threshold to manage the transmission power of the femtocell base station. In another work, SINR is derived based on the distance calculation model and used with the capacity in order to arrange the radio resources among various femtocells, so unwanted interference can be managed [22]. In [23], a proposed scheme also computes the achieved SINR for UEs and generates an emergency message, which coordinates the communication between macrocell and femtocell to control the femtocell transmission power near a particular macrocell’s UE which experienced interference from the nearby femtocell. In [24], estimated SINR is calculated and used for two basic methods: PC-1 and PC-2. Both methods were introduced to reduce the undesirable impact of the interference based on controlling the transmission power in terms of the downlink.

Furthermore, femtocells can be clustered into different clusters based on certain criteria. Such an approach divides the radio resources according to the resulting clustering mechanism and assigns them among femtocells. This will ensure avoidance of the interference among femtocells where adjacent femtocells would be assigned different radio resources. In [25], a model is first used to identify the interference victim and aggressor. Then, the SCVC mechanism categorized UEs based on their status: whether it is critical or not. As an afterthought, the scheme would cluster femtocells into various clusters based on the status of their UEs. Accordingly, the radio resources would be assigned among clusters where co-tier interference can be managed. In [26], the authors divided the problem into two sub-problems. The LINGO was used as a basis to propose a mathematical model for the clustering problem. Moreover, a novel scheme was proposed to assign sub-channels among UEs of the femtocells. The authors in [27] proposed a mechanism for joint clustering and resource allocation. According to their approach, maximizing the throughput is the objective of their modeled optimization problem. The clustering of the femtocells is considered in order to address the interference challenge. An approach called data-driven power control (DDPC) was introduced in [28]. The DDPC scheme uses the affinity propagation (AP) clustering algorithm to cluster various femtocells based on collected data, such as reference signal received power (RSRP), for every femtocell’s UEs. A cluster approach was also considered in [29]. In this, the line of sight connectivity was considered to cluster femtocells and their UEs to overcome two main challenges. Such challenges were co-tier interference and performance loss. In [30], the temperature of the interference among femtocells was used to cluster femtocells into multiple clusters. The proposed mechanism operates in two steps, one for assigning radio resources and the other for controlling the power where the interference could be mitigated.

The femtocells’ coverage and their radius may play a major role in addressing some challenges of deploying femtocells with the coexistence of a macrocell. The transmission power of certain femtocells can be adjusted with the maximum level to extend the coverage. However, the maximum transmission power of a particular femtocell may cause interference to its adjacent femtocells. Therefore, managing the coverage for femtocells can be used to alleviate the undesirable impact of interference and improve the network’s capacity. In [31], the authors proposed a consensus-based distributed coverage mechanism. In this work, controlling the transmission power, which influences the coverage of the femtocell base station, was considered to improve the performance of the system and achieve fairness among femtocells’ UEs. Additionally, coverage optimization was considered in [32]. The proposed framework evolved in different aspects. It aimed to support the self-organization network (SON) approach. In [33], the femtocell coverage would be controlled based on the received signals’ quality, which would be received from adjacent femtocells. The goal was to achieve a high level of fairness among UEs while exchanging less information. A bargaining game was used in [34] in order to enhance the coverage of femtocells. The framework also developed a computationally efficient approach to improve the performance of the overall network. Furthermore, small cells are categorized in [35] based on coverage into overlapped and non-overlapped coverage. The authors proposed this framework to optimize the implementation of WiFO, a hybrid femtocell architecture based on Wi-Fi and free-space optical (FSO). The objective was to improve the throughput of the network.

## 3. System Model and Problem Formulation

### 3.1. System Model

In this system, a dense co-channel implementation of the femtocell network is considered. The system involves a heterogeneous network according to LTE technology. This system consists of femtocell networks that are installed in indoor environments. Femtocells are also connected to each other through an entity, which is called femto-gateway (FGW). Increasing cell capacity is anticipated by deploying more femtocells. The performance of the system is evaluated based on predefined calculations.

Accordingly, the SINR for UEs attached to macrocell MUEs and for UEs attached to femtocell FUEs is estimated based on the calculation of the path loss. Path loss is formed in different models, such as outdoor path loss and indoor path loss. For the outdoor MUEs, the path loss is given by [36]:

(1) $$PathL_{indB}=15.3+37.6\;log_{10}\;\left(D\right)$$

Additionally, a path loss for the indoor MUEs is given as follows:

(2) $$PathL_{indB}=15.3+37.6\;log_{10}\;\left(D\right)\;+\;L_{outW}$$

where the distance in meters between transmitter and receiver is denoted as (D), and the penetration loss generated by outdoor wall is $L_{outW}$. Moreover, we can calculate path loss between the femtocell and its attached FUEs based on the following formula [36]:

(3) $$\begin{array}{c} PathL_{indB}=38.46+20log_{10}\;\left(D\right)\;+\;0.7d_{2R,\;indoor}\\ +\;18.3n^{\left((n+2)/(n+1)-0.46\right)}\;+\;W_{x}\;*\;L_{inW} \end{array}$$

where the number of walls that separate the femtocell base station and its associated FUEs is $W_{x}$, and the penetrated floors are accounted for as *n*. Walls that separate apartments would cause penetration loss, which is $L_{i}nw$, while the penetration loss, which is caused by walls inside the apartments, is accounted for in meters and is $0.7d_{2R,\;indoor}$.

The following formula is used in order to calculate the path loss of the indoor femtocell and associated FUEs who are positioned in an outdoor environment [36]:

(4) $$\begin{array}{c} PathL_{indB}=max(15.3+37.6\;log_{10}\;\left(D\right),\\ 38.46+20log_{10}\;\left(D\right))\;+\;0.7d_{2R,\;indoor}+\\ 18.3n^{\left((n+2)/(n+1)-0.46\right)}+\;W_{x}\;*\;L_{inw}\;+\;L_{outW} \end{array}$$

The throughput of the system is evaluated through determining a received SINR from macrocell UE *i* on subcarrier *s* with interference received from neighbor macrocells as well as nearby femtocells. Accordingly, SINR can be estimated as follows [37]:

(5) $$SINR_{i,s}=\frac{P_{m,s}\;G_{i,m,s}}{\sum_{m^{\prime }}P_{m^{\prime },s}G_{i,m^{\prime },s}\;+\;\sum_{F}P_{F,s}\;G_{i.F,s}+\;N_{0}\Delta f}$$

where $G_{i,m,s}$ is the channel gain between serving macrocell *m* and MUE *i* on subcarrier *s*, and $P_{m,s}$ is the transmitting power for serving macrocell *m* on subcarrier *s*. $P_{m^{\prime },s}$ is the transmitting power for neighboring macrocells $m^{\prime }$ on subcarrier *s*. $G_{i,m^{\prime },s}$ is the channel gain between neighboring macrocells $m^{\prime }$ and MUE *i* on subcarrier *s*. $G_{i.F,s}$ $P_{F,s}$ is the transmitting power for neighboring femtocell *F* on subcarrier *s*. $G_{i.F,s}$ is the channel gain between neighboring femtocell *F* and MUE *i* on subcarrier *s*. Δf is the subcarrier spacing, and $N_{0}$ is the white noise power spectral density.

The interference received from the serving macrocell and all neighboring femtocells is considered in order to estimate the SINR received from FUE *j* on subcarrier *s*, given by [37]:

(6) $$SINR_{j,s}=\frac{P_{F,s}\;G_{j,F,s}}{\sum_{m}P_{m,s}G_{j,m,s}\;+\;\sum_{F^{\prime }}P_{F^{\prime },s}\;G_{j.F^{\prime },s}+\;N_{0}\Delta f}$$

where $P_{F,s}$ the transmitting power for serving femtocell *F* on subcarrier *s*, and $G_{j,F,s}$ the channel gain between serving femtocell *F* and FUE *j* on subcarrier *s*. $P_{m,s}$ is the transmitting power for serving macrocell *m* on subcarrier *s*. $G_{i,m,s}$ is the channel gain between serving macrocell *m* and FUE *j* on subcarrier *s*. $P_{F^{\prime },s}$ is the transmitting power for neighboring femtocell $F^{\prime }$ on subcarrier *s*. $G_{j.F^{\prime },s}$ is the channel gain between neighboring femtocell $F^{\prime }$ and FUE *j* on subcarrier *s*.

Whether the UE is positioned outdoor or indoor, the channel gain calculation is given as [37]

(7) $$G=10^{-PathL/10}$$

Accordingly, the capacity of UE *u* on subcarrier *s* can be expressed as follows [37]:

(8) $$C_{u,s}=\Delta f\;log_{2}\left(1\;+\;\alpha SINR_{u,s}\right)$$

where the target BER is α, and $10^{-6}$ is set to be considered in this work as BER.

The system comprises of *K* femtocell base stations, which are implemented and overlaid on a particular macrocell coverage, and they are all sharing the same bandwidth *B*. The bandwidth *B* mainly consists of *N* sub-channels. Every femtocell base station has a number of femtocell UEs that is *U*.

Assume $p_{k,u,n}$ specifies the allocated transmission power for a particular sub-channel *n* for femtocell user *u* attached to femtocell *k*, where n∈[1,2,3,4,5,…,N], u∈[1,2,,3,4,5,…,U], and k∈[1,2,3,4,5,…,K]. The power allocation matrix of certain *K* femtocell base stations is indicated by $P={\left[p_{k,u,n}\right]}_{K\times U\times N}$. Moreover, the sub-channel allocation indicator matrix is indicated by $A={\left[a_{k,u,n}\right]}_{K\times U\times N}$.

When sub-channel *n* is allocated to the femtocell’s UE *u* in femtocell base station *k*, $a_{k,u,n}=1$; otherwise, $a_{k,u,n}=0$.

Now, the capacity of sub-channel *n* that is allocated to the femtocell’s UE *u* in femtocell base station *k* can be calculated as follows [37]:

(9) $$C_{k,u,n}=\Delta f\;log_{2}\left(1\;+\;\alpha SINR_{k,u,n}\right)$$

### 3.2. Problem Formulation

The transmission power constraint is considered for the femtocell base station *k* when transmitting its radio resources (sub-channels) $N_{k}$; ensuring that the transmission power will not exceed the predefined maximum transmission power $P_{maximum}$, the constraint is determined as follows:

(10) $$\sum_{n=1}^{N}\;a_{k,u,n}p_{k,u,n}\leq P_{maximum},\;\forall k,u.$$

In addition, maintaining the performance of the system essentially depends on considering QoS requirement $q_{u}$ for UE *u*:

(11) $$\sum_{n=1}^{N}\;a_{k,u,n}C_{k,u,n}\geq q_{u},\;\forall k,u.$$

To constrain the unfavorable impact of the interference, an interference level and limit are enforced. Assume $I_{n}$ to be maximum limit of interference on sub-channel *n*:

(12) $$\sum_{u=1}^{U}\sum_{k=1}^{K}\;a_{k,u,n}p_{k,u,n}G_{k,u,n}\leq I_{n},\;\forall n.$$

A sub-channel *n* is required to be assigned for only a single femtocell’s UE *u* at a time. Thus, a consideration of the following constraint is made:

(13) $$\sum_{u=1}^{U}\;a_{k,u,n}\leq 1,\;\forall k,n.$$

Based on the given constraints of QoS and interference, the target is formed to maximize the overall capacity. As a result, the associated problem can, therefore, be formulated as follows:

(14a) $$\underset{a_{k,u,n},p_{k,u,n}}{\mathrm{maximize}}\sum_{k=1}^{K}\sum_{u=1}^{U}\sum_{n=1}^{N}a_{k,u,n}C_{k,u,n}$$

(14b) $$\begin{array}{l} \mathrm{subject}\;\mathrm{to}\\ \sum_{u=1}^{U}\sum_{n=1}^{N}a_{k,u,n}p_{k,u,n}\leq P_{maximum},\;\forall k,u \end{array}$$

(14c) $$p_{k,u,n}\;\geq \;0\;\;\forall k,u,n$$

(14d) $$\sum_{n=1}^{N}\;a_{k,u,n}C_{k,u,n}\geq q_{u},\;\forall k,u$$

(14e) $$\sum_{u=1}^{U}\sum_{k=1}^{K}\;a_{k,u,n}p_{k,u,n}G_{k,u,n}\leq I_{n},\;\forall n$$

(14f) $$\sum_{u=1}^{U}\;a_{k,u,n}\leq 1,\;\forall k,n$$

(14g) $$a_{k,u,n}\in \left[0,1\right],\;\forall k,u,n.$$

where (14b) is considered to constrain the transmission power for each femtocell base station *k*; (14c) indicates positive and non-negative power transmission; (14d) maintains the QoS; (14e) indicates the tolerable interference temperature for each sub-channel *n*; (14f) and (14g) ensure that each sub-channel *n* is assigned only to a single UE *u* in each femtocell base station *k*.

The following section describes the proposed scheme. The proposed scheme encompasses two stages: radio resource allocation and controlling the transmission power.

- Radio Resource Allocation Scheme
- Power Control Scheme

## 4. Proposed Scheme

### 4.1. Radio Resource Allocation

#### 4.1.1. Coverage and Spectrum Division

The frame structure in LTE is 10 ms in terms of time domain. The frame is divided into 10 subframes where each subframe equals 1 ms. In addition, a particular subframe contains two slots where each slot is 0.5 ms. Seven or six o orthogonal frequency division multiple access (OFDMA) symbols are contained in each slot. In LTE, the radio resource is structured as 2D resource blocks (RBs) where one RB is considered as the smallest unit that can be scheduled for a particular UE. A single RB represents 12 subcarriers. The bandwidth of a single RB is 180 KHz in a single time slot. The UE would first request RBs from the base station. Accordingly, the UE would be considered for scheduling based on the scheduling strategy. The number of assigned RBs to a single UE is varied according to multiple factors. Therefore, a scheduling strategy should be designed carefully in order to achieve the preferred performance objectives. There are different scheduling strategies and algorithms, which control the process of distributing the radio resources among attached UEs. In this work, we consider the round robin strategy. Moreover, the bandwidth in LTE can be 1.4 MHz, 3 MHz, 5 MHz, 10 MHz, or 20 MHz. The number of RBs is varied based on the chosen bandwidth. For example, 20 MHz can provide 100 RBs for each scheduling run.

The first step of the proposed scheme is to arrange the distribution of the available radio resources between the macrocell and femtocells, which are installed in the macrocell’s coverage area. Therefore, the concept of fractional frequency reuse (FFR) is adopted [20]. The main idea behind the FFR is to partition the macrocell’s coverage into two major sub-areas: center Center(C) area and edge area Edge(E). Then, the edge area would be partitioned into three different sub-areas E1,E2,E3. Accordingly, the spectrum would be divided into four sub-bands (A,B,C,D). The divided sub-bands would be distributed among four different sub-areas in terms of macrocell UE aspects *A* to *C*, *B* to E1, *C* to E2, and *D* to E3. On the other hand, femtocell UEs, which are positioned in the same sub-area alongside macrocells’ UEs, would be assigned different sub-bands. For example, sub-bands A,C,D would be allocated for the femtocell’s UEs, which are positioned in sub-area E1, while macrocells’ UEs are assigned sub-band *B*. In these scenarios, femtocells’ UEs and macrocell’s UEs positioned in the same sub-area can operate in different subcarriers. Accordingly, the cross-tier interference can be alleviated and managed.

However, adjacent femtocells may interfere with each other. Consequently, a co-tier interference problem would be experienced. Therefore, this scheme is proposed to distribute the available sub-channel for a group of femtocells, which are positioned in a particular sub-area, in such a way that co-tier interference can be mitigated.

Thus, a group of sub-bands that are considered for femtocells, which are installed in a particular sub-area, would be reunited and channelized according to femtocells’ density. Consequently, an interference-based table is constructed for channelizing the dedicated sub-band purpose. This table is also necessary for the second stage of this scheme. The second stage of this scheme manages the transmission power for femtocells and addresses the problem of co-tier interference.

#### 4.1.2. Construction the Interference-Based Table

We assume that *N* is the number of femtocells that are implemented in sub-area $X_{i}$ where $N_{1}^{n}\;=\;[f_{1},f_{2},f_{3},f_{4},$……$,f_{n}]$. Multiple sub-bands are contained in a set $W_{i}$. $W_{i}$ will be divided into multiple channels according to the given *N*, so $W_{i}\;=\;[CL_{1},CL_{2},CL_{3},CL_{4},$……$,CL_{n}]$. Consequently, every $CL_{i}$ would be assigned to a particular femtocell $f_{i}$. The core idea behind this channelizing procedure is to differentiate among sub-channels in order to adjust transmission power. Transmission power can be adjustable with constraint of not exceeding the maximum threshold. The strategy for adjusting the transmission power will be discussed in the second stage.

The interference conflict among the neighboring femtocells influences constructing the interference-based table. Each femtocell $f_{i}\in N_{1}^{n}$ would recognize $I_{i}$ set. This $I_{i}$ set includes all femtocells that cause interference to femtocell $f_{i}$. Table 2 depicts all femtocell base stations that are placed in a particular sub-area and all their correspondent *I* sets.

A predefined constant domain $\lambda_{x}$ is considered where femtocell base stations of set *I* can be specified. Each femtocell $f_{i}$ has its private constant domain $\lambda_{i}$, which is used to determine femtocells of set $I_{i}$, which produces interference to femtocell $f_{i}$. Hence, $\lambda_{i}$ is modeled as follows:

(15) $$\lambda_{i}\;\geq \;\pi \left({\left(\alpha \;*\;r_{i}\right)}^{2}\right)$$

where $r_{i}$ is the radius of femtocell $f_{i}$, and α is a constant defined and given based on:

(16) α≥2.5

Accordingly, the femtocell can sense the neighboring femtocells based on the Equation (16), where the constant value of α can be predetermined and must not be less than the double coverage of a certain femtocell. Furthermore, each femtocell can be aware of and recognize all sources of interference. Moreover, their sub-channels also know, which facilitates the process of interference management. Table 3 depicts all femtocells in the system with their divided sub-channels. This step is necessary to recognize the set of sub-channels that is transmitted with adjustable transmission power. Consequently, a combination between Table 2 and Table 3 is used to characterize sub-channels and identifies sub-channels that need to be transmitted with adjustable transmission power or transmitted with maximum transmission power. The process of adjusting the transmission power for certain sub-channels is given in this scheme’s second stage.

According to Table 2, a set of $I_{i}$, which is constructed for each femtocell $f_{i}$, represents the femtocells that cause interference to femtocell $f_{i}$. This data is integrated with the data given in Table 3 in order to produce Table 4. Each row of Table 2 will be combined with a corresponding row of Table 3. Then, identical femtocells’ ID in both sets will be identified. Consequently, any channel $CL_{i}$ dedicated to femtocell $f_{i}$, which presents in set *I*, will be marked. Hence, the adjustable transmission power for a certain sub-channel can be determined and considered.

The interference conflict map is depicted in Table 4, generated from Table 2 and Table 3. This table’s production is produced by mapping interference information in Table 2 with channel information in Table 3. If there is a marked sub-channel $CL_{i}$ in Table 4, it means that $f_{x}$ introduces interference to $f_{i}$. This channel $CL_{i}$ is associated with the column of $f_{x}$. Consequently, femtocell $f_{i}$ included in the first row of Table 3 would run and utilize all available sub-channels $CL_{1}\ldots to\ldots CL_{n}$. The sub-channels that are marked as $CL_{i}*$ would be transmitted with adjustable transmission power according to the second stage of this scheme.

**Figure 2:**
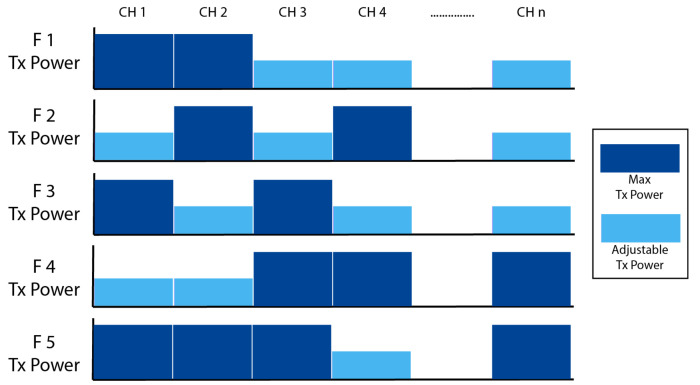
Channel with Max Tx power and adjustable Tx power.

Figure 2 gives an example and depicts an illustration where various sub-channels can be distinguished, whether they will be transmitted with maximum transmission power or adjustable transmission power. For example, $f_{2}$ can operate and use all sub-channels $CL_{1}\ldots to\ldots CL_{n}$. Accordingly, sub-channels $CL_{2}$ and $CL_{4}$ will be transmitted with maximum transmission power, while sub-channels $CL_{1}$ and $CL_{3}$ will use the proposed mechanism, which is explained in the second stage of this scheme, to adjust the transmission power. The next subsection provides more details about the second stage of this scheme, where the transmission power of some sub-channels is adjusted in order to alleviate the undesirable impact of the co-tier interference among femtocell base stations. The pseudo-code for the radio resource allocation strategy is illustrated in Algorithm 1.
**Algorithm 1** Radio Resource Allocation1:$\;N_{1}^{n}\;=\;\left[f_{1}\;,\;f_{2},\;f_{3},\ldots \;f_{n}\right]$2:Bandwith=[A,B,C,D]3:$M_{C}\;\leftarrow \;A;\;M_{E1}\;\leftarrow \;B;\;M_{E2}\;\leftarrow \;C;\;M_{E3}\;\leftarrow \;D$4:$F_{C}\;\leftarrow \;\left[B+C+D\right];\;F_{E1}\;\leftarrow \;\left[A+C+D\right];\;F_{E2}\;\leftarrow \;\left[A+B+D\right];\;F_{E3}\;\leftarrow \;\left[A+B+C\right]$5:$f_{i}\;\leftarrow F_{x}/N_{x}$6:**while**i≤N**do**7:      **for**j=1 to *N* **do**8:        **if** $\;D_{i,j}\;\leq \lambda \;\&\&\;i\;\neq \;j\;$ **then**9:            $I_{i}\;\leftarrow \;f_{j}$10:        **end if**11:    **end for**12:**end while**

### 4.2. Power Control Strategy

In this subsection, a proposed strategy for controlling some sub-channels’ transmission power is introduced and explained. All available radio resources would be used to improve the system’s capacity. Nevertheless, using all radio resources would introduce an interference challenge. An unfavorable impact of interference would decrease throughput. However, controlling the transmission power for the same sub-channels received from different femtocells could alleviate the co-interference’s undesirable impact. Hence, a strategy of controlling transmission power should be considered to overcome the challenge of the negative impact of interference.

The proposed strategy accredits femtocell base stations to adjust their transmission power autonomously. The ultimate aim of this scheme is to alleviate undesirable interference in the co-channel deployment process. In the previous sub-section, the radio resource allocation strategy was introduced, which is the necessary step for the scheme’s second stage. Table 2 would also be used in the controlling transmission power strategy.

#### Interference Level Calculation

Essentially, Table 2 would be reformed to construct and derive the interference’s temperature, which is caused by each femtocell $f_{i}$. This procedure is essential and prerequisite in order to model and define an appropriate cost function. Substantially, by using an interference-based table, we can recognize the frequency amount for each femtocell $f_{x}$, which is reported in sets of *I* for the whole system (from $I_{1}\ldots$ to $\ldots I_{n}$). The main idea is to realize the level of unfavorable interference impact introduced by each femtocell $f_{i}$ in the system and assign an adequate cost function.

Now, we generate and construct a new set Θ. This set of Θ contains all *I* sets (from $I_{1}$ to $I_{n}$) for all femtocell base stations (from $f_{1}$ to $f_{n}$). Subsequently, we need to check all frequencies of certain femtocell $f_{i}$ in the collection of sets $I_{1\ldots n}$, which is stored in set Θ. Thereafter, each femtocell $f_{i}$ can determine its $\theta_{i}$. Accordingly, $\theta_{i}$ reports the amount of frequencies of femtocell $f_{i}$ in Θ. This signalizes the number of other femtocells, which are influenced by femtocell $f_{i}$. Table 5 shows the process of forming Θ and $\theta_{i}$.

Accordingly, $\theta_{i}$ evolves the frequencies of each femtocell $f_{i}\;\in \;N_{1}^{n}$ that happens in Θ. Therefore, $\theta_{i}$ includes the total amount of femtocells interfered with and impacted by $f_{i}$ in the predefined coverage model as $\lambda_{i}$, which is defined by Equations (15) and (16).

A maximum transmission power limit is identified for all femtocell base stations as $Tx_{\left[Max\right]}$. Femtocells have the ability to autonomously adjust transmission power for each sub-channel under interference constraint. Some sub-channels might be transmitted with maximum transmission power $Tx_{\left[Max\right]}$, while the rest of the sub-channels would be transmitted with adjustable transmission power with consideration of interference. According to the previous subsection, each femtocell can realize the sub-channels that need to be transmitted with maximum transmission power or adjustable transmission power.

The level of suitable transmission power is varied from one femtocell to another. The cost function $\sigma_{i}$, which is derived for each femtocell $f_{i}$, influences the level of adjustable transmission power for each femtocell $f_{i}$. Once the cost function $\sigma_{i}$ rate is high, the level of the transmission power of its femtocell needs to be decreased. The total number of femtocells that are interfered by femtocell $f_{i}$ influences the level of its cost function $\sigma_{i}$ rate. The rate would be derived and constructed from $\theta_{i}$ set under the constraint of $\lambda_{i}$ domain. Hence, the rate of cost function $\sigma_{i}$ of certain femtocell base station $f_{i}$ is modeled as follows:

(17) $$\sigma_{i}=\frac{\theta_{i}}{FBS_{total}}$$

where $FBS_{total}$ is the total number of femtocells, which are installed in a certain sub-area *X*. Equation (17) is used as an essential component of Equation (18) because it reflects the temperature of undesirable interference, and it is necessary for determining the adequate transmission power where the interference would be seen.

Ultimately, each femtocell $f_{i}$ can autonomously set an adequate amenable transmission power for some predefined sub-channels. This scheme allows each femtocell $f_{i}\;\in \;N_{1}^{n}$ to determine its suitable adjustable transmission power scale according to the interference amount that it introduces to other nearby femtocells within the $\lambda_{i}$ domain. Accordingly, the adjustable downlink transmission power level for a group of sub-channels, which is transmitted by femtocell $f_{i}\;\in \;N_{1}^{n}$, is given as follows:

(18) $$Tx_{i}=Tx_{\left[Max\right]}\;-\;\left[Tx_{\left[Max\right]}\;*\;\sigma_{i}\right]$$

### 4.3. Proposed Scheme Summary

Table 6 summarized the previous discussion. All significant processes of the scheme are summarized in Table 6. All processes would be performed in order to allow femtocells to determine an adequate level of downlink transmission power. Accordingly, interference could be managed and mitigated. Moreover, Equation (18) might be utilized for configuring the downlink transmission power for the predefined smallest sub-channels or for the entire allocated sub-band for a particular femtocell. The pseudo-code for autonomously adjusting downlink transmission power is given in Algorithm 2.
**Algorithm 2** Tx power control.1:$N_{1}^{n}\;=\;\left[f_{1}\;,\;f_{2},\;f_{3},\ldots \;f_{n}\right]$2:$FBS_{total}\;=\;n$3:$I_{1}^{n}\;=\phi$4:$\Theta_{1}^{n}\;=\;\left[I_{1},\;I_{2},\;I_{3},\ldots \;I_{n}\right]$5:**while**i≤n**do**6:     **while** j≤n **do**7:         **if** $\;D_{i}\;\leq \lambda_{i}\;\&\&\;i\;\neq \;j\;$ **then**8:            $I_{i}\;\leftarrow \;f_{j}$9:         **end if**10:     **end while**11:**end while**12:**while**x≤n**do**13:     **for** y≤n **do**14:         **if** Θ(x,y)==x&&x≠y **then**15:            $\theta_{x}\leftarrow \;\theta_{x}\;+\;1$;16:         **end if**17:     **end for**     $\sigma_{x}\leftarrow \;\frac{\theta_{x}}{FBS_{total}}$     $Tx_{x}\;=\;\left(Tx_{\left[max\right]}\;-\;\left(Tx_{\left[max\right]}\;*\;\sigma_{x}\right)\right)$18:**end while**

## 5. Simulation Results and Discussion

In this section, the results and simulation parameters are presented. The performance of the proposed scheme is evaluated using a simulation. The MATLAB tool is used for building the simulation setup. The system model, which is provided early in this work, is considered for this system. In this simulation, an LTE-based heterogeneous network is assumed. The OFDMA transmission scheme is considered for macrocell and incorporated femtocells. The frequency band is split into subcarriers. A set of twelve consecutive subcarriers form a single RB, which is the smallest unit that can be assigned to a particular UE. In addition, the available spectrum is autonomously divided into four sub-bands. Each sub-band is allocated to a certain sub-area of a macrocell after dividing the macrocell into four sub-areas. Each sub-band contains a particular number of RBs. The number of RBs in each sub-band depends on the channel bandwidth. In this work, the channel bandwidth is assumed as 20 MHz, which contains 100 RBs. All available RBs are assigned for femtocells where the proposed scheme has the ability to alleviate the interference.

The UEs of the macrocell are distributed randomly. The UEs of the macrocell would be offloaded to nearby femtocells. The channel condition for each UE is unique and differs from other UEs. Multiple factors can impact the channel condition. Noise, density of femtocells, SINR, and penetration loss would not be the same for all UEs.

**Figure 3:**
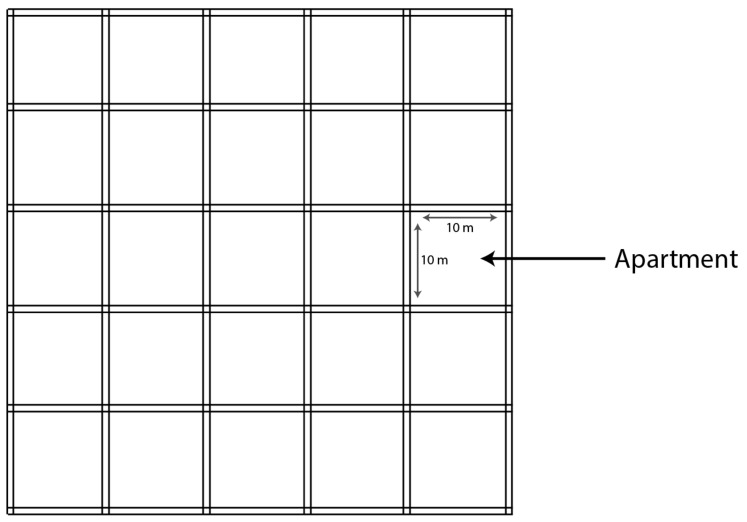
5 × 5 grid model, reproduced from [38].

The model of deploying femtocells is assumed as depicted in Figure 3. Indeed, Figure 3 depicts the grid model of 5 × 5 [38], which is adopted in this work. It contains a building of 25 adjacent apartments. The area of each apartment is considered as 100 m${}^{2}$. Femtocell base stations are positioned at the center of each apartment. At least a single UE is associated with each femtocell. The femtocell base stations are assumed to be placed in an indoor environment. At least one FUE is attached to a particular femtocell in the indoor environment. The number of femtocells is sequentially increased. While the number of femtocells is increasing, the macrocell offloads more UEs to femtocells in order to increase the capacity. Table 7 depicts the main assumptions that are made in this work. The simulation parameters are summarized in Table 7. For instance, the coverage of a certain femtocell is assumed to be 5 m, which is assumed to be adequate for the apartment’s size.

**Figure 4:**
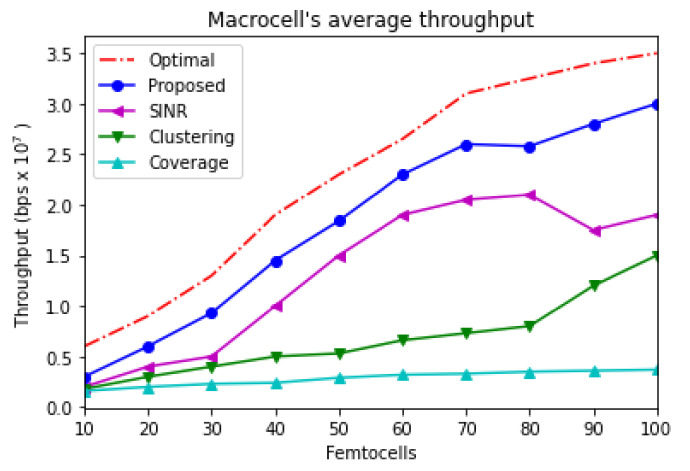
Macrocell average throughput.

Figure 4 depicts the average throughput of the overall network. In each run, the number of inserted femtocells is changed every run. Furthermore, UEs, which are associated with macrocells, are offloaded onto femtocells when they are inserted into the system. Hence, the capacity is increased once the number of femtocells is increased. The proposed mechanism is compared with three different approaches: clustering-based, SINR-based, and coverage-based. The clustering-based approach distributes femtocells among different clusters in order to allocate the radio resources among these clusters. It separates the femtocells into different clusters and allocates radio resources among clusters. The goal of the system is to ensure that identical sub-channels would not be assigned to adjacent femtocells in order to address the co-interference challenge. This allocation strategy ensures that neighboring clusters operate in different radio resources. Therefore, the number of clusters would be increased with the dense deployment of femtocells. As a result, the dedicated spectrum portion for each cluster would be decreased. Accordingly, the interference issue would be addressed. However, the overall capacity of the network would be affected. The SINR-based approach is used to control the transmission power of the femtocell based on the received SINR. The transmission power is adjusted according to the received SINR from the attached UEs. In this, the transmission power of certain femtocells is set dynamically. Therefore, the femtocell network capacity is affected by any change occurring to the SINR value for any reason. Additionally, the dense deployment of femtocells influences SINR for attached UEs. As a result, the overall capacity of the network might be degraded. The coverage-based approach coordinates between serving macrocells and femtocells in order to adjust the transmission power of the femtocell. The coverage of the femtocell base station would be determined based on its position and its distance from the macrocell. This can be achieved by examining the received signal strength from the macrocell. Accordingly, each femtocell base station would received a signal from its nearby macrocells. The femtocell transmission power is adjusted based on the received signal strength in order to avoid interference with MUEs.

According to the given information from Figure 4, the worst capacity is achieved by the coverage-based approach. The clustering-based gains better throughput than the coverage-based approach due to its efficiency of allocating radio resources among femtocells so interference can be managed and controlled. However, the clustering-based scheme performs worse than SINR-based and the proposed schemes. The SINR-based scheme performs better than coverage-based and clustering-based schemes. However, as the figure illustrates, SINR-based is not suitable when the number of femtocells is increased. The proposed scheme illustrates better performance among the other three schemes because it includes two strategies: radio resources allocation strategy and power control strategy. As the figure depicts, the capacity is increased dramatically when more femtocells are inserted into the system. For example, the network’s capacity with 100 femtocells is almost double the network’s capacity when the number of femtocells becomes 50 because traffic is offloaded from the macrocell onto femtocells. The proposed scheme provides almost similar results with 50 femtocells and 60 femtocells because the simulation randomly distributes the femtocells, and at this stage, it may densely locate femtocells close to each other. This becomes more clear with the SINR-based scheme when femtocells, which are located close to each other, receive SINR from a short distance. Furthermore, the clustering-based scheme depicts consistency because it allocates different RBs for adjacent femtocells. However, the clustering scheme could not control the transmission power.

**Figure 5:**
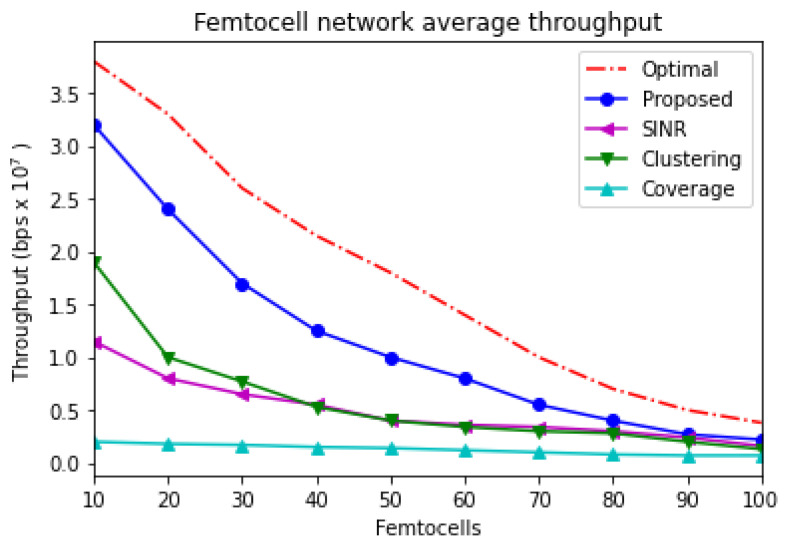
Femtocell network average throughput with interference consideration.

Additionally, the average throughput of the femtocell network is illustrated in Figure 5. The number of inserted femtocells is incremented periodically. In this figure, only the femtocell network is considered. Therefore, the average throughput is decreased when more femtocells are inserted into the system because of the unfavorable impact of co-interference. Indeed, the system’s performance would be degraded when femtocell base stations are deployed densely in certain areas. The performance of the SNIR-based and clustering-based schemes changes when the number of inserted femtocells is changed. Accordingly, the SINR-based scheme would perform better than the clustering-based scheme when the inserted femtocells are increased. However, the clustering-based scheme prefers a lower number of deployed femtocells. Moreover, the clustering-based scheme illustrates worse performance once the number of installed femtocells is increased. In addition, the worst performance is achieved by a coverage-based scheme because it follows a strategy that does not realize resource allocation and power control approaches. However, the proposed scheme jointly considers both approaches. As a result, the proposed scheme achieves the best performance compared to other schemes. According to the figure, the throughput of all schemes is decreased because of the interference impact. As the number of adjacent femtocells, which are densely deployed, is increased, the impact of co-interference would be severe.

**Figure 6:**
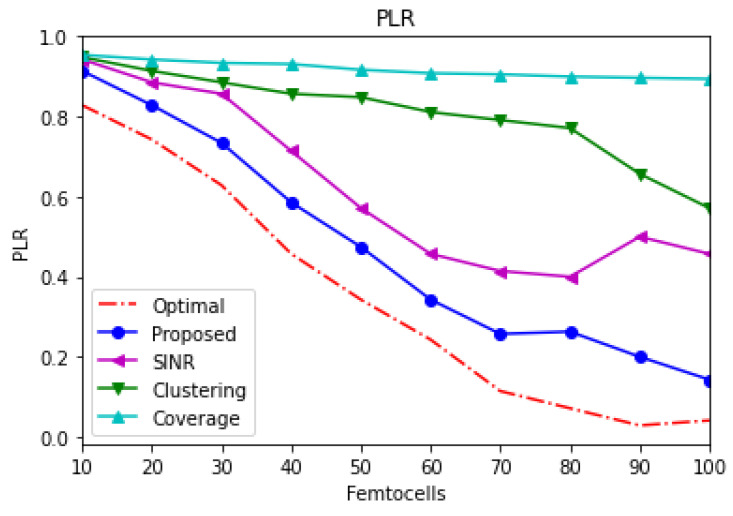
Macrocell average packet loss.

Figure 6 depicts the average packet loss of the overall network, including the macrocell and different numbers of inserted femtocells. The coverage-based scheme delivers the worst average of the packet loss, while both proposed and SINR-based schemes show the best average packet loss compared to other schemes. The clustering-based scheme delivers median results compared to other schemes. In addition, the average packet loss is improved when more femtocells are inserted into the system.

## 6. Conclusions

Femtocell deployment should increase the overall capacity of heterogeneous networks. However, dense deployment of femtocells might raise several challenges. Cross-tier and co-tier interferences are considered to be one of the significant challenges. In this work, a scheme consisted of two phases proposed to mitigate the undesirable impact of interference. Hence, the dense deployment of femtocells could be controlled. The first stage of the scheme was an allocation strategy used to dedicate sub-bands to four different sub-areas of the macrocell extent. Then, the sub-bands could be further channelized based on the femtocell density in a particular sub-area. Afterward, each femtocell could realize sub-channels that increase the unfavorable impact of the interference. In the second stage, a transmission power control mechanism was introduced, where the co-tier interference could be managed. The transmission power of a particular femtocell could be adjusted autonomously with consideration of the interference to improve the network’s overall capacity. The proposed mechanisms attempt to preserve an acceptable level of network capacity as much as possible. Additionally, the proposed scheme was simulated and compared with three different schemes. The proposed scheme showed the best performance among the compared schemes. The optimal solution requires very high complexity. The proposed solution provides low complexity, where the complexity of Algorithm 1 is $O(n^{2})$, and the complexity of Algorithm 2 is $O(n^{4})$.

The evaluation of the scheme has been done through a conducted simulation. However, it should be evaluated in a real-world experimental environment. The real-world experimental environment would show realistic results as well as apparent weaknesses of the proposed scheme. Therefore, we will consider a real-world experimental environment in future work. The proposed scheme provides the ability to use all available radio resources alongside resolving the issue of co-interference. This ability is the main strength of the proposed mechanism.

## Figures and Tables

**Table 1:** Summary of the interference types.

Secnario	Source	Victim	Interference’s Type	Transmission Mode
M-UE-to-F-BS	M-UE	FBS	Cross-tier	Uplink
M-BS-to-F-UE	MBS	F-UE	Cross-tier	Downlink
F-UE-to-M-BS	F-UE	MBS	Cross-tier	Uplink
F-BS-to-M-UE	FBS	M-UE	Cross-tier	Downlink
F-BS-to-F-UE	FBS	F-UE	Co-tier	Downlink
F-UE-to-F-BS	F-UE	FBS	Co-tier	Uplink

**Table 2:** Femtocells with their corresponding set of *I*.

FBS	Set *I*
$f_{1}$	$f_{a_{1}},f_{a_{2}},f_{a_{3}},\ldots \ldots ,f_{a_{m}}$
$f_{2}$	$f_{b_{1}},f_{b_{2}},f_{b_{3}},\ldots \ldots ,f_{b_{m}}$
$f_{3}$	$f_{c_{1}},f_{c_{2}},f_{c_{3}},\ldots \ldots ,f_{c_{m}}$
$f_{4}$	$f_{d_{1}},f_{d_{2}},f_{d_{3}},\ldots \ldots ,f_{d_{m}}$
. .	……
. .	……
. .	……
. .	……
. .	……
. .	……
$f_{n}$	$f_{e_{1}},f_{e_{2}},f_{e_{3}},\ldots \ldots ,f_{e_{m}}$

**Table 3:** Channelizing band based on femtocells density.

	$f_{1}$	$f_{2}$	$f_{3}$	$f_{4}$	...	$f_{n}$
$f_{1}$	$CL_{1}$	$CL_{1}$	$CL_{1}$	$CL_{1}$	……	$CL_{1}$
$f_{2}$	$CL_{2}$	$CL_{2}$	$CL_{2}$	$CL_{2}$	……	$CL_{2}$
$f_{3}$	$CL_{3}$	$CL_{3}$	$CL_{3}$	$CL_{3}$	……	$CL_{3}$
$f_{4}$	$CL_{4}$	$CL_{4}$	$CL_{4}$	$CL_{4}$	……	$CL_{4}$
. .	……	……	……	……	……	……
. .	……	……	……	……	……	……
. .	……	……	……	……	……	……
. .	……	……	……	……	……	……
. .	……	……	……	……	……	……
. .	……	……	……	……	……	……
$f_{n}$	$CL_{n}$	$CL_{n}$	$CL_{n}$	$CL_{n}$	……	$CL_{n}$

**Table 4:** Interference-based table.

	$f_{1}$	$f_{2}$	$f_{3}$	$f_{4}$	...	$f_{n}$
$f_{1}$	$CL_{1}$	$CL_{1}*$	$CL_{1}$	$CL_{1}*$	……	$CL_{1}$
$f_{2}$	$CL_{2}$	$CL_{2}$	$CL_{2}*$	$CL_{2}*$	……	$CL_{2}$
$f_{3}$	$CL_{3}*$	$CL_{3}*$	$CL_{3}$	$CL_{3}$	……	$CL_{3}$
$f_{4}$	$CL_{4}*$	$CL_{4}$	$CL_{4}$	$CL_{4}$	……	$CL_{4}*$
. .	……	……	……	……	……	……
. .	……	……	……	……	……	……
. .	……	……	……	……	……	……
. .	……	……	……	……	……	……
. .	……	……	……	……	……	……
. .	……	……	……	……	……	……
$f_{n}$	$CL_{n}*$	$CL_{n}*$	$CL_{n}$	$CL_{n}$	……	$CL_{n}$

**Table 5:** Constructing Θ set.

FBS	Θ	$\Theta^{\prime }$
$f_{1}$	$f_{x_{1}},f_{x_{2}},f_{x_{3}},\ldots \ldots .,f_{x_{m}}$	$\theta_{1}$
$f_{2}$	$f_{x_{1}},f_{x_{2}},f_{x_{3}},\ldots \ldots .,f_{x_{m}}$	$\theta_{2}$
$f_{3}$	$f_{x_{1}},f_{x_{2}},f_{x_{3}},\ldots \ldots .,f_{x_{m}}$	$\theta_{3}$
$f_{4}$	$f_{x_{1}},f_{x_{2}},f_{x_{3}},\ldots \ldots .,f_{x_{m}}$	$\theta_{4}$
. .	……	. .
. .	……	. .
. .	……	. .
. .	……	. .
. .	……	. .
. .	……	. .
$f_{n}$	$f_{x_{1}},f_{x_{2}},f_{x_{3}},\ldots \ldots .,f_{x_{m}}$	$\theta_{n}$

**Table 6:** Autonomous Tx power process.

FBS	Θ	$\Theta^{\prime }$	σ	${\mathrm{Tx}}_{i}$
$f_{1}$	$f_{x_{1}},f_{x_{2}},\ldots \ldots ,f_{x_{m}}$	$\theta_{1}$	$\sigma_{1}=\frac{\theta_{1}}{FBS_{total}}$	$Tx_{1}=Tx_{\left[Max\right]}\;-\;\left[Tx_{\left[Max\right]}\;*\;\sigma_{1}\right]$
$f_{2}$	$f_{x_{1}},f_{x_{2}},\ldots \ldots ,f_{x_{m}}$	$\theta_{2}$	$\sigma_{2}=\frac{\theta_{2}}{FBS_{total}}$	$Tx_{2}=Tx_{\left[Max\right]}\;-\;\left[Tx_{\left[Max\right]}\;*\;\sigma_{2}\right]$
$f_{3}$	$f_{x_{1}},f_{x_{2}},\ldots \ldots ,f_{x_{m}}$	$\theta_{3}$	$\sigma_{3}=\frac{\theta_{3}}{FBS_{total}}$	$Tx_{3}=Tx_{\left[Max\right]}\;-\;\left[Tx_{\left[Max\right]}\;*\;\sigma_{3}\right]$
$f_{4}$	$f_{x_{1}},f_{x_{2}},\ldots \ldots ,f_{x_{m}}$	$\theta_{4}$	$\sigma_{4}=\frac{\theta_{4}}{FBS_{total}}$	$Tx_{4}=Tx_{\left[Max\right]}\;-\;\left[Tx_{\left[Max\right]}\;*\;\sigma_{4}\right]$
. .	……	. .	..	..
. .	……	. .	..	..
. .	……	. .	..	..
. .	……	. .	..	..
. .	……	. .	..	..
. .	……	. .	..	..
$f_{n}$	$f_{x_{1}},f_{x_{2}},\ldots \ldots ,f_{x_{m}}$	$\theta_{n}$	$\sigma_{n}=\frac{\theta_{n}}{FBS_{total}}$	$Tx_{n}=Tx_{\left[Max\right]}\;-\;\left[Tx_{\left[Max\right]}\;*\;\sigma_{n}\right]$

**Table 7:** Simulation Parameters.

Parameters	Value
Macrocell Radius ($r_{m}$)	500 m
Femtocell Radius ($r_{f}$)	5 m
Frequency	2 GHz
Macrocell Transmission Power $P_{m}$	46 dBm
Maximum Femtocell Transmission Power $P_{f,max}$	21 dBm
Outdoor Walls Loss $L_{ow}$	15 dB
Indoor Walls Loss $L_{iw}$	7 dB
Bandwidth	20 MHz
Subcarrier Spacing	15 kHz
White Noise Power Density	−174 dBm/Hz
Traffic Model	Full Buffer

## Data Availability

Data sharing not applicable.
